# Rapid visual detection and differentiation of canine and feline parvovirus via a one-tube RPA-CRISPR/Cas13a assay

**DOI:** 10.3389/fmicb.2025.1735549

**Published:** 2025-12-19

**Authors:** Zhurui Shao, Wenkai Zhang, Changbin Jin, Zhibo Yang, Jingyi Yang, Xiaohu Zhang, Xintong Huang, Yufei Yang, Ruizi Ren, Yiwen Zhang, Jieen Weng, Yueping Zhang, Hao Shi

**Affiliations:** 1 State Key Laboratory of Veterinary Public Health and Safety, College of Veterinary Medicine, China Agricultural University, Beijing, China; 2 China Agricultural University Veterinary Teaching Hospital, Beijing, China

**Keywords:** CRISPR/Cas13a, rapid visual detection, canine parvovirus type 2, feline parvovirus, RPA, VP2 gene

## Abstract

Canine parvovirus type 2 (CPV-2) and feline parvovirus (FPV) are highly contagious pathogens that pose significant threats to domestic and wild carnivores. Rapid and accurate diagnosis is crucial for outbreak control and wildlife conservation, especially in resource-limited settings. In this study, we developed a one-tube RPA-CRISPR/Cas13a platform for the rapid detection and differentiation of CPV-2 and FPV. Two systems were established: a universal detection system for simultaneous identification of both viruses, and a differential detection system to distinguish between them. By targeting conserved regions of the VP2 gene and optimizing reaction conditions, we achieved high sensitivity and specificity. The universal system exhibited a limit of detection (LOD) of 10^2^ copies/μL, while the differential system reached 10^3^ copies/μL, with both assays completed within 60 min. Clinical validation using 50 samples showed 100% concordance with q-PCR and sequencing results. This study established a dual detection system that is sensitive, rapid, and suitable for use in primary-level settings and field conditions. It holds significant application value in enhancing the early diagnosis and differentiation of canine and feline parvoviruses, reducing the risk of transmission, and protecting the health of wildlife.

## 1 Introduction

Canine parvovirus type 2 (CPV-2) and feline parvovirus (FPV) belong to the genus *Parvovirus* within the family *Parvoviridae* (Decaro and Buonavoglia, 2012). These non-enveloped viruses exhibit icosahedral symmetry, with a diameter ranging from 20 to 24 nm (Nelson et al., 2008; Decaro and Buonavoglia, 2012). The genomes of CPV-2 and FPV are single-stranded DNA, approximately 5,300 bp in length, and contain two open reading frames (ORFs) that encode structural proteins (VP1 and VP2) and non-structural proteins (NS1 and NS2) (Decaro and Buonavoglia, 2012). The VP2 protein, a major component of the parvoviral capsid, plays a critical role in maintaining the structural integrity of the virus, facilitating the infection process, and determining the host range (Parker and Parrish, 1997; Carreira et al., 2004; Nelson et al., 2008). Both CPV-2 and FPV not only infect domestic dogs and cats but also circulate widely in wildlife populations, posing threats to species such as cheetahs, Siberian tigers, raccoons, and giant pandas (Thalwitzer et al., 2010; Wünschmann et al., 2021; Kimpston et al., 2022; Yeo et al., 2023; Zhao et al., 2023). Highly contagious and environmentally resistant, CPV-2 and FPV are transmitted via the fecal-oral route, primarily targeting the intestinal tract, bone marrow, and immune system, which can result in severe gastroenteritis, immunosuppression, and high mortality rates (Steinel et al., 2001).

CPV-2 is closely related to FPV, exhibiting high genetic sequence homology. Since its emergence, CPV-2 has undergone multiple evolutionary events, continuously giving rise to new antigenic variants (Chen et al., 2021). Initially, the VP2 protein of CPV-2 differed from that of FPV by only six amino acids, and CPV-2 was considered a host-adapted variant of FPV, that was incapable of infecting cats (Truyen, 1999; Zhou et al., 2017). However, with the spread and recombination of CPV-2 in dogs, it was gradually replaced by its antigenic variants (CPV-2a, CPV-2b, and CPV-2c), and several reports have shown that some of these variants can infect certain felids (Truyen, 2006; Decaro et al., 2010). Allison et al. analyzed the phylogenetic history of carnivore parvoviruses based on 234 complete VP2 gene sequences and found that viruses from the same host species were broadly distributed on the phylogenetic tree, suggesting that these viruses underwent multiple independent cross-species transmission events. Notably, virial sequences from raccoons and pumas were identified in both CPV-like and FPV-like clusters, further supporting the hypothesis that CPV-2 variants and FPV possess the capacity for cross-species transmission (Allison et al., 2013). In 2018, Ahmed et al. simultaneously detected CPV-2 and FPV in rectal swabs from 40 diarrheic dogs collected in Pakistan (Ahmed et al., 2018). In 2024, Behera et al. reported that CPV-2 and FPV were detected by PCR in 38 suspected-infected cats in India (Behera et al., 2024). The cross-species transmission of CPV-2 and FPV may enhance viral adaptability through mutation and recombination, potentially facilitating the emergence of novel variants capable of spreading among wildlife populations, threatening their health, and contributing to population declines. Therefore, the development of diagnostic tools capable of simultaneously detecting and differentiating CPV-2 and FPV is essential for early diagnosis, timely isolation of infected animals, containment of outbreaks, and the reduction of associated economic losses.

Currently, various methods are employed for the detection of CPV-2 and FPV, including virus isolation followed by immunofluorescence assay (IFA), immunoassays such as lateral flow tests and enzyme-linked immunosorbent assay (ELISA), as well as nucleic acid-based techniques, including conventional PCR and quantitative PCR (q-PCR) (Digangi et al., 2011; Meggiolaro et al., 2017; Minakshi et al., 2016; Zou et al., 2022). Although these methods offer accurate detection of CPV-2 and FPV, they are operationally complex, time-intensive, and necessitate specialized personnel and equipment, thereby limiting their applicability for rapid diagnosis in field settings or resource-constrained environments. Recent advancements in Recombinase Polymerase Amplification (RPA) and Clustered Regularly Interspaced Short Palindromic Repeats (CRISPR) technologies have led to the development of integrated RPA-CRISPR-based detection assays (Ahamed et al., 2024; Jiang et al., 2024; Chen et al., 2025). RPA is an isothermal nucleic acid amplification method that operates at 37–42 °C without the need for drastic temperature changes, allowing efficient target amplification within 5–20 min (Munawar, 2022). The CRISPR-Cas13a system, a Class 2 Type VI system, is an RNA-guided RNA nuclease that specifically targets and degrades RNA (Makarova et al., 2020). In 2017, Zhang Feng’s team integrated the CRISPR-Cas13a system with RPA to develop the Specific High-sensitivity Enzymatic Reporter unLOCKing (SHERLOCK) detection platform, which has proven invaluable in molecular diagnostics (Kellner et al., 2019). Subsequently, RPA-CRISPR/Cas13a-based assays have been successfully applied to detect pathogens such as Severe Acute Respiratory Syndrome Coronavirus 2 (SARS-CoV-2), Ebola virus, Dengue virus (DENV), and Zika virus (ZIKV) (Barnes et al., 2020; Crone et al., 2020; Li et al., 2020).

This study developed a detection method based on RPA and CRISPR/Cas13a technologies. By targeting conserved regions of the VP2 gene in CPV-2 and FPV, crRNA and RPA primers were designed to establish two distinct detection and differentiation of CPV-2 and FPV. This method is rapid, sensitive, and does not require complex equipment, offering a practical and field-deployable diagnostic approach for monitoring CPV-2 and FPV outbreaks and supporting wildlife protection efforts, especially in resource-constrained environments.

## 2 Materials and methods

### 2.1 Sample collection and detection

We collected 56 clinical samples from the China Agricultural University Veterinary Teaching Hospital, including 25 canine-origin samples from dogs suspected of having canine parvovirus infection, 25 feline-origin samples from cats suspected of having feline parvovirus infection, and one confirmed positive sample each for canine distemper virus, canine coronavirus, canine parainfluenza virus, feline coronavirus, feline calicivirus, and feline herpesvirus. Detailed information is provided in attachment. According to the instructions provided with the Taq Pro U + Multiple Probe q-PCR Mix (Nanjing Vazyme Biotech Co., Ltd), the q-PCR reaction system was prepared as follows: 10 μL of 2 × Taq Pro U + Multiple Probe q-PCR Mix, 1 μM each of forward and reverse primers, 250 nM TaqMan Probe Mix, 0.4 μL of 50 × ROX Reference Dye 2, and 1 μL of template DNA. The remaining volume was adjusted with DNase/RNase-free deionized water to a final volume of 20 μL. The results obtained from this setup were used as the standard reference results. In addition, each sample was subjected to DNA sequencing (Sangon Biotech) to confirm whether the pathogen was CPV-2 or FPV.

### 2.2 Construction of positive plasmid standards

A total of 50 VP2 gene sequences were obtained from the GenBank database, comprising 10 representative sequences from each of the following variants: CPV-2, CPV-2a, CPV-2b, CPV-2c, and FPV,^1^ and sequence alignment was performed using Snapgene 6.1.1. Conserved regions were selected as target gene sites for the RPA-CRISPR/Cas13a system nucleic acid detection. The VP2 gene figments of CPV-2 and FPV were synthesized and cloned into the pUC57-Kan plasmid by Sangon Biotech (Shanghai) Co., Ltd. to serve as positive controls and quantitative standards (Supplementary Table S1).

### 2.3 Expression and purification of Cas13a protein

The pCold-TF-Ulp1-SUMO-LwCas13a expression plasmid (Cas13a derived from *Leptotrichia wadei*), previously constructed in our laboratory, was transformed into *E. coli* BL21 (DE3) competent cells. Protein expression was induced with 500 μM IPTG at 18 °C for 18 h. Harvested cells were lysed using high-pressure homogenization, and the clarified lysate was filtered and purified via Ni-affinity chromatography using a HisTrap HP column (Cytiva lifesciences). Eluted fractions were analyzed by SDS-PAGE, and the target protein was further purified by cation-exchange chromatography. Final buffer exchange to storage buffer (500 mM NaCl, 50 mM Tris–HCl, pH 7.5, 5% glycerol, 2 mM DTT) was performed using centrifugal ultrafiltration. Protein concentration was measured with a BCA assay, aliquoted (50 μL), snap-frozen in liquid nitrogen, and stored at −80 °C.

### 2.4 Design and preparation of RPA primers, crRNA, and RNA reporter

**Figure 1:**
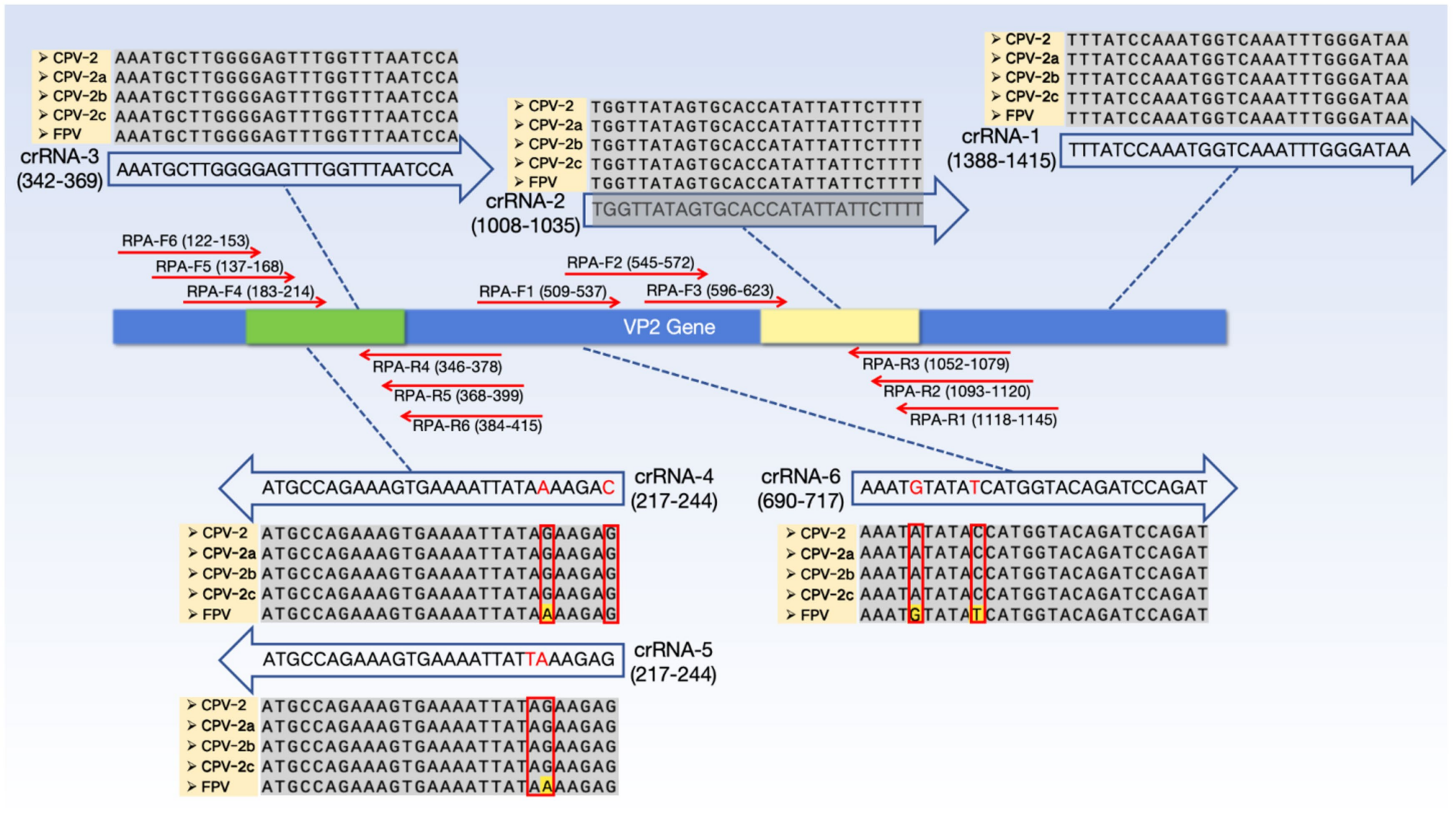
In the RPA-CRISPR/Cas13a detection system, the positions of crRNA and RPA primer pairs are as follows: crRNA-1, crRNA-2, and crRNA-3 are used in the universal detection system (capable of specifically detecting CPV-2 and FPV), while crRNA-4, crRNA-5, and crRNA-6 are used in the differentiation detection system (able to distinguish between CPV-2 and FPV). RPA-F1, RPA-F2, RPA-F3, RPA-R1, RPA-R2, and RPA-R3 are used to amplify the crRNA target sites with the highest detection efficiency in the universal detection system, whereas RPA-F4, RPA-F5, RPA-F6, RPA-R4, RPA-R5, and RPA-R6 are used to amplify the crRNA target sites with the highest detection efficiency in the differentiation detection system. crRNA-4, crRNA-5, and crRNA-6 are annotated with mismatch information for CPV-2 and FPV. crRNA-4 and crRNA-5 are derived from the same original crRNA sequence, which has no mismatches with FPV but contains a single mismatch at position 6 with CPV-2. We intentionally introduced an additional mismatch at position 1 to generate crRNA-4, and another mismatch at position 7 to generate crRNA-5. crRNA-6 is a different crRNA sequence without any artificial modifications; it has two mismatches with CPV-2 at positions 5 and 10, but no mismatches with FPV. The arrows of the crRNAs and RPA primers, respectively, indicate their directions. The yellow regions on the VP2 gene represent the target amplification areas for the universal detection system, while the green regions indicate the target amplification areas for the differentiation detection system. The sequences of CPV-2 (OR257446.2), CPV-2a (KY073269.1), CPV-2b (KX774252.1), CPV-2c (OR230516.1), and FPV (OR667804.1) shown in the figure are derived from GenBank.

Based on the alignment results in section 2.2, three crRNAs (crRNA-1, crRNA-2, and crRNA-3) targeting highly conserved regions of the VP2 gene were designed for the simultaneous detection of CPV-2 and FPV (Figure 1). When designing crRNAs to differentiate CPV-2 from FPV, previous studies have indicated that the central region of the crRNA-target RNA duplex, known as the “seed region” is sensitive to mismatches (Abudayyeh et al., 2016). We differentiated one region in the VP2 gene with two nucleotide mismatches between CPV-2 and FPV, and another region with a single mismatch, to which an additional artificial mismatch was introduced. As a result, three crRNAs with distinct mismatch patterns (crRNA-4, crRNA-5, and crRNA-6) were designed. Details of the mismatch sites are shown in Figure 1. The crRNAs were synthesized via *in vitro* transcription. Full-length crRNA primers containing the T7 promoter were annealed and used as templates for RNA transcription. The transcription was performed overnight at 37 °C using a T7 high-yield transcription kit (Nanjing Vazyme Biotech Co., Ltd). The resulting transcripts were purified using an RNA purification kit (Zymo Research), and the crRNA concentration was measured with a Nano-Drop 2000c (Thermo Fisher Scientific). The purified crRNAs were aliquoted and stored at −80 °C.

Based on the optimization experiments, two crRNAs with the highest detection efficiency were selected. Three pairs of RPA primers were designed in the conserved regions upstream and downstream of each crRNA target site. According to the orientation of the crRNA sequence, the T7 promoter was introduced into either the upstream or downstream primer to ensure that the transcribed target RNA after amplification would be complementary to the crRNA. The RNA reporter was labeled with FAM at the 5′ end and BHQ1 at the 3′ end. All RPA primers, crRNAs, and RNA reporter sequences were synthesized by GENEWIZ. The sequences used in this study are listed in Supplementary Table S2.

### 2.5 RPA reaction

The RPA (Recombinase Polymerase Amplification) reaction was performed using the TwistAmp® Liquid Basic kit according to the manufacturer’s instructions with minor modifications. Each 12.5 μL RPA reaction contained the following components: 6.25 μL of 2 × Reaction Buffer, 0.9 μL of 25 mM dNTPs, 1.25 μL of 10 × E-mix, 0.6 μL each of forward and reverse RPA primers, 0.625 μL of 20 × Core Reaction Mix, 0.5 μL of DNA template, and 1.15 μL of DNase/RNase-free deionized water. Finally, 0.625 μL of MgOAc was added to initiate the reaction. The reaction mixture was incubated at 37 °C for 15 min to allow for amplification. After the RPA reaction, the amplification products need to be heated at 65 °C for 10 min to inactivate the proteins in the RPA system, allowing the proteins to separate from the amplified DNA and preventing the formation of smearing bands.

### 2.6 Establishment of the CRISPR/Cas13a reaction system

The CRISPR/Cas13a reaction system was established using target RNAs transcribed and purified from PCR-amplified pUC57-CPV and pUC57-FPV plasmids. For the detection of CPV-2 and FPV, the reaction mixture contained 45 nM Cas13a protein, 22.5 nM crRNA, 125 nM RNA reporter, 1 μL RNase inhibitor, 1.5 μL NEB r3.1 buffer, and 1 μL of target RNA. RNase-free ddH_2_O was added to adjust the final volume to 20 μL. For differentiating between CPV-2 or FPV, the concentrations of key components were increased: Cas13a protein was used at 450 nM, crRNA at 225 nM, and ssRNA reporter at 1.25 μM. The remaining components and reaction volume were the same as described above. The mixture was incubated in a Real-time PCR System (Applied Biosystems) at 37 °C for 30 min, with fluorescence signals collected every 1 min.

### 2.7 Establishment of one-tube RPA-CRISPR/Cas13a detection method

A one-tube RPA-CRISPR/Cas13a detection system was developed using pUC57-CPV and pUC57-FPV plasmids as target DNA templates. For the detection of CPV-2 and FPV, the reaction mixture contained: 10 μL of 2 × Reaction Buffer, 25 mM dNTPs, 2 μL of 10 × E-mix, 0.48 μM forward primer, 0.48 μM each of forward and reverse primers, 20 × Core Reaction Mix, 0.5 μL T7 RNA Polymerase Mix, 0.5 μL RNase inhibitor, 25 mM rNTPs, 45 nM Cas13a protein, 22.5 nM crRNA, 125 nM ssRNA reporter, and 1.5 μL NEB Buffer r3.1, then, 1 μL of target DNA and 14 mM MgOAc were added to the tube cap (without mixing). The total volume was adjusted to 20 μL. After a brief centrifugation to combine all components, the reaction was incubated in a Real-time PCR System (Applied Biosystems) at 37 °C for 90 min, with fluorescence signals collected every 1 min. To further quantify the limit of detection, we analyzed the endpoint fluorescence signals of the detection system and evaluated the data from blank controls. The lower limit of detection (LLD) was defined as the lowest concentration at which the signal exceeded the mean of the blank control plus three standard deviations. RNase-free water was used as the template in the blank controls, and the values of X_blank and SD_blank were obtained from three independent replicates conducted using both the universal detection assay and the differential detection assay.

### 2.8 Optimization of the reaction system

The endpoint fluorescence value output by the Real-time PCR System was used as the basis for optimizing the RPA-CRISPR/Cas13a fluorescence detection reaction. The optimization process included the selection of the most efficient crRNA and RPA primers. Additionally, key reaction parameters were systematically evaluated and optimized, including reaction time, temperature, Cas13a protein concentration, crRNA concentration, RNA reporter concentration, and the volume of RNase-free water used to dilute the reaction system.

### 2.9 Limit of detection and specificity analysis

The copy numbers of the constructed pUC57-CPV-2 and pUC57-FPV plasmids were calculated, and a 10-fold serial dilution was performed using RNase-free ddH_2_O to achieve concentrations ranging from 10^5^ to 10^1^ copies/μL. For each concentration, 1 μL of the diluted plasmid was used as the template in the RPA-CRISPR/Cas13a reaction. The endpoint fluorescence intensity was measured after 60 min of reaction to evaluate the sensitivity of the detection system. To assess the specificity of the RPA-CRISPR/Cas13a detection system, nucleic acids extracted from several other common pathogens of dogs and cats [including Canine distemper virus (CDV), Canine coronavirus (CCV), Canine parainfluenza virus (CPIV), Feline coronavirus (FCoV), Feline calicivirus (FCV), and Feline herpesvirus-1 (FHV-1)] were used as templates in the RPA-CRISPR/Cas13a reaction. Real-time fluorescence signals were recorded by Real-time PCR System (Applied Biosystems), and the endpoint fluorescence values were collected at 60 min to evaluate cross-reactivity and specificity.

### 2.10 Statistical analysis

Each reaction was performed in triplicate, and data were analyzed using GraphPad Prism version 10.3.1. A one-way ANOVA was conducted to evaluate the significance of differences among multiple groups for a single variable, while two-way ANOVA were used to compare differences between two groups. Data are presented as the mean ± standard deviation (SD). Asterisks (***) denote highly significant differences between groups (*p* < 0.001).

## 3 Results

### 3.1 Establishment of a one-tube RPA-CRISPR/Cas13a detection method

**Figure 2:**
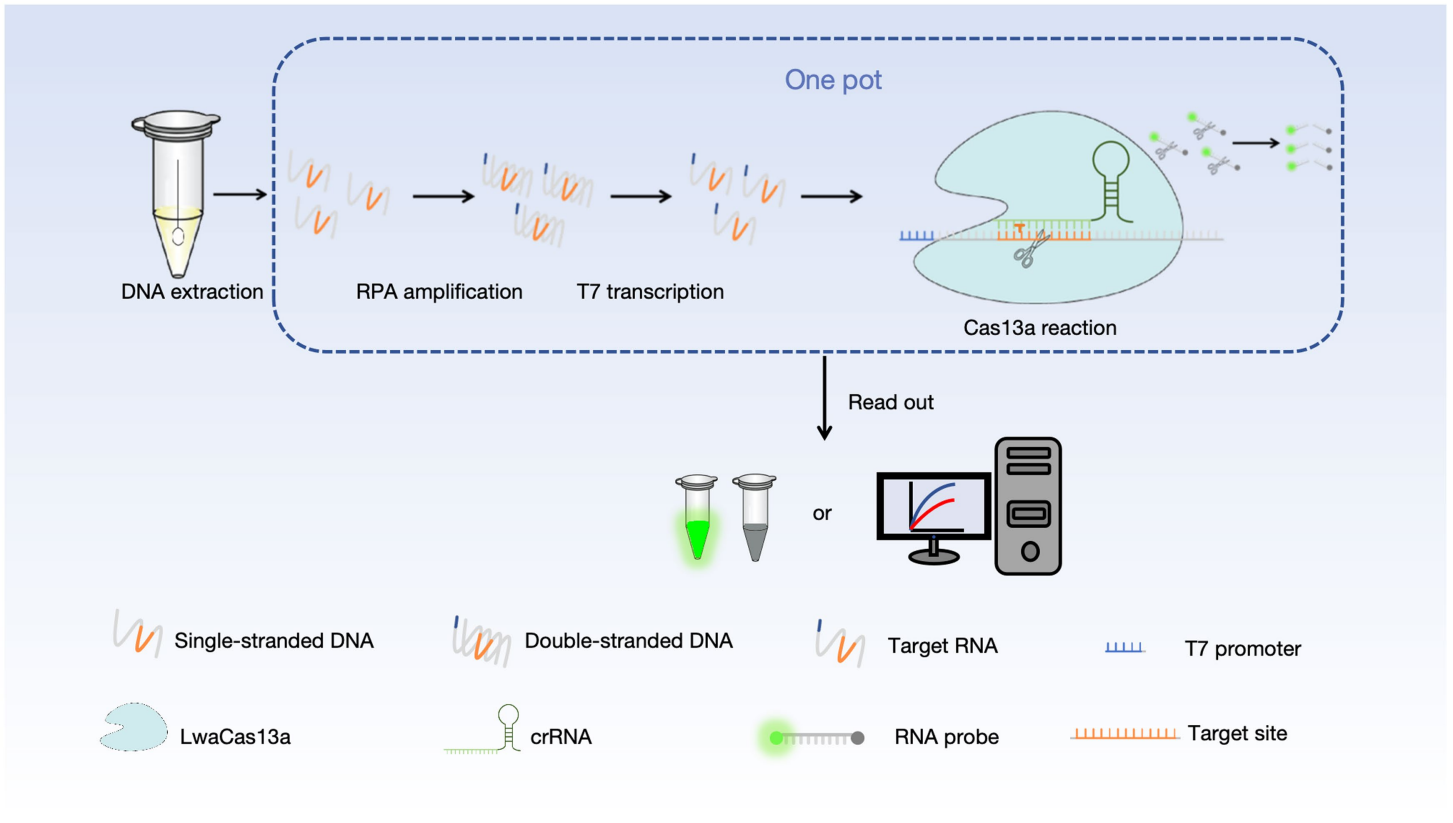
The workflow of RPA-CRISPR/Cas13a detection. Viral nucleic acids are extracted from the samples using a automated nucleic acid extractor. The DNA target is then amplified via RPA and subsequently transcribed into an RNA target using T7 transcription. The crRNA-Cas13a complex binds to the amplified RNA target, triggering the trans-cleavage activity of Cas13a, which leads to the cleavage of the RNA reporter gene. The cleaved RNA reporter gene can be visualized either by direct observation under blue light or through fluorescence signal detection.

To establish a rapid and accurate method for the detection and differentiation of CPV-2 and FPV, we developed two one-tube RPA-CRISPR/Cas13a-based assays with high sensitivity and specificity. As shown in Figure 2, viral nucleic acids were extracted from clinical samples using an automated nucleic acid extraction system and served as templates for the RPA-CRISPR/Cas13a reaction. In the presence of target sequences, the products amplified by RPA and transcribed by T7 RNA polymerase are recognized by the Cas13a-crRNA complex, which activates the trans-cleavage activity of the Cas13a protein. This leads to the cleavage of a single-stranded RNA (ssRNA) reporter labeled with FAM at the 5′ end and BHQ1 at the 3′ end, generating a green fluorescence signal that can be visually observed under blue light or quantitatively measured using a fluorescence detector. The detection workflow first utilizes a universal detection system to determine whether the sample is infected with CPV-2 or FPV. If the result is positive, a differential detection system is then employed to distinguish between CPV-2 and FPV infections.

### 3.2 Selection of the optimal crRNA and RPA primer pairs

**Figure 3:**
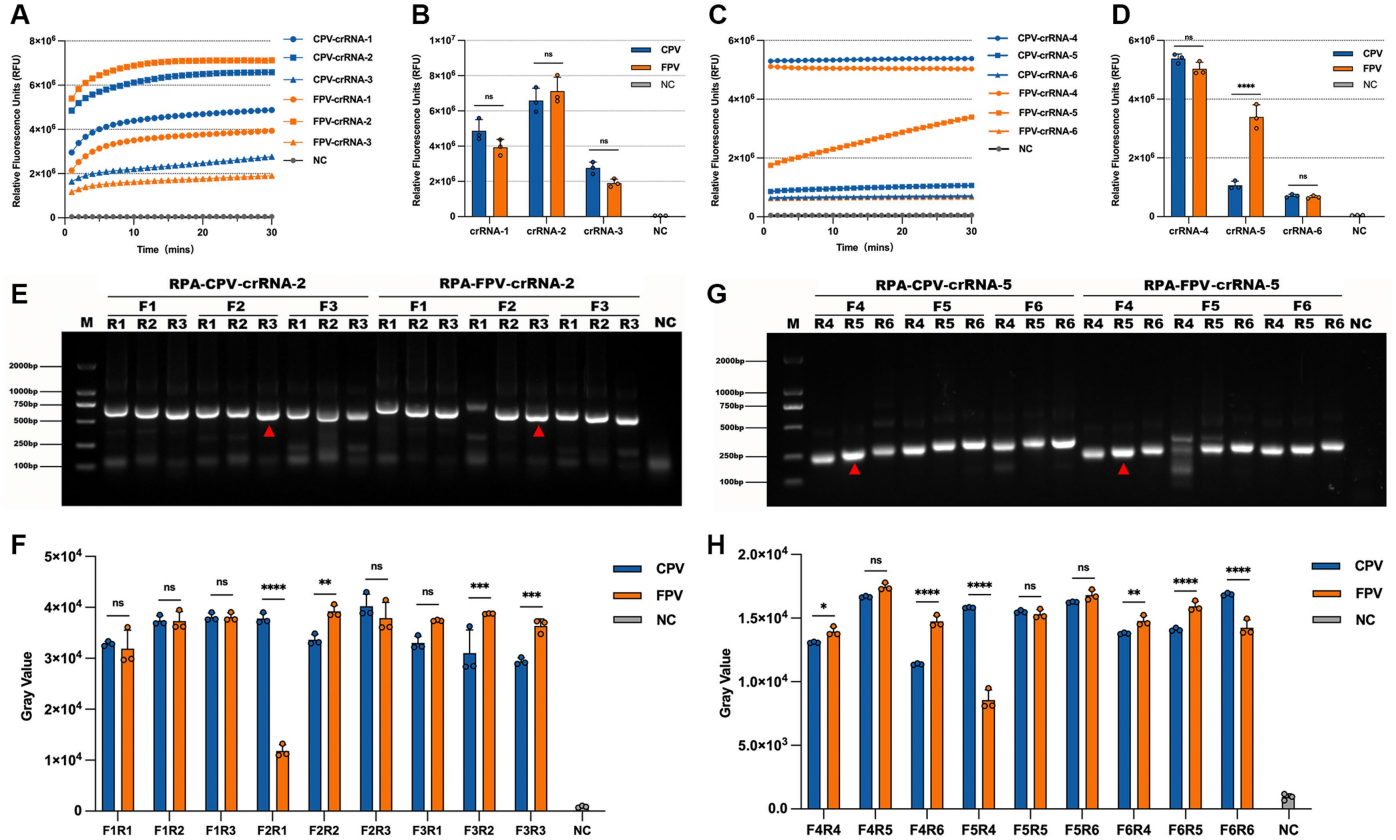
Selection of optimal crRNAs and RPA primers. CRRNA-1, CRRNA-2, CRRNA-3, CRRNA-4, CRRNA-5, and CRRNA-6 were evaluated in the CRISPR/Cas13a reaction based on fluorescence kinetics at 30 min **(A,C)** and endpoint fluorescence signals **(B,D)**. For the CRRNA-2 target, three pairs of upstream and downstream primers were designed, forming nine different combinations. RPA amplification was performed using pUC57-CPV and pUC57-FPV plasmids as templates, and the results were analyzed by gel electrophoresis **(E)**. Grayscale intensity of the gel images was analyzed using ImageJ, with three independent replicates, and the results are shown as bar graphs in **(F)**. Similarly, for the CRRNA-5 target, three primer pairs were designed, also forming nine combinations. RPA amplification using pUC57-CPV and pUC57-FPV plasmids as templates was analyzed through gel electrophoresis, with grayscale intensity quantified from three independent experiments **(G)**, and corresponding bar graphs shown in **(H)**. Nuclease-free water was used as the template in negative controls. ***p < 0.001. Error bars represent standard deviation.

To identify optimal guide sequences for CRISPR/Cas13a-based detection, three candidate crRNAs (crRNA-1, crRNA-2, and crRNA-3) were designed and evaluated for the simultaneous detection of CPV-2 and FPV. All three crRNAs demonstrated robust activity, with fluorescence intensities significantly higher than the no-target control (NTC). Among them, crRNA-2 yielded the highest fluorescence signal at 30 min and reached peak intensity more rapidly than the other candidates, thereby being selected for subsequent universal detection assays (Figures 3A,B). Similarly, three additional crRNAs (crRNA-4, crRNA-5, and crRNA-6) were assessed for their ability to differentiate between CPV-2 and FPV. crRNA-4 produced strong fluorescence signals in the presence of both viral templates, whereas crRNA-6 failed to generate a signal significantly above that of the NTC, rendering it unsuitable for differential detection. Notably, crRNA-5 generated a strong fluorescence response with FPV but not with CPV-2, indicating its specificity for FPV. Therefore, crRNA-5 was selected for use in the differentiation assay between CPV-2 and FPV (Figures 3C,D).

To optimize the RPA reaction, three pairs of primers were designed for both the detection and differentiation systems, resulting in nine primer combinations tested for each. Using pUC57-CPV and pUC57-FPV plasmids as templates, RPA amplification was performed and evaluated by agarose gel electrophoresis to assess amplification efficiency. In the universal detection system, the primer pair RPA-F2/RPA-R3, targeting the crRNA-2 recognition region, exhibited the highest amplification efficiency and robust performance for both CPV-2 and FPV (Figures 3E,F), and was therefore selected for subsequent detection experiments. In the differentiation system, the RPA-F4/RPA-R5 pair also showed the highest amplification efficiency and strong performance for both CPV-2 and FPV (Figures 3G,H), and was chosen for further applications in distinguishing between CPV-2 and FPV.

### 3.3 Optimization of the CRISPR/Cas13a reaction conditions

**Figure 4:**
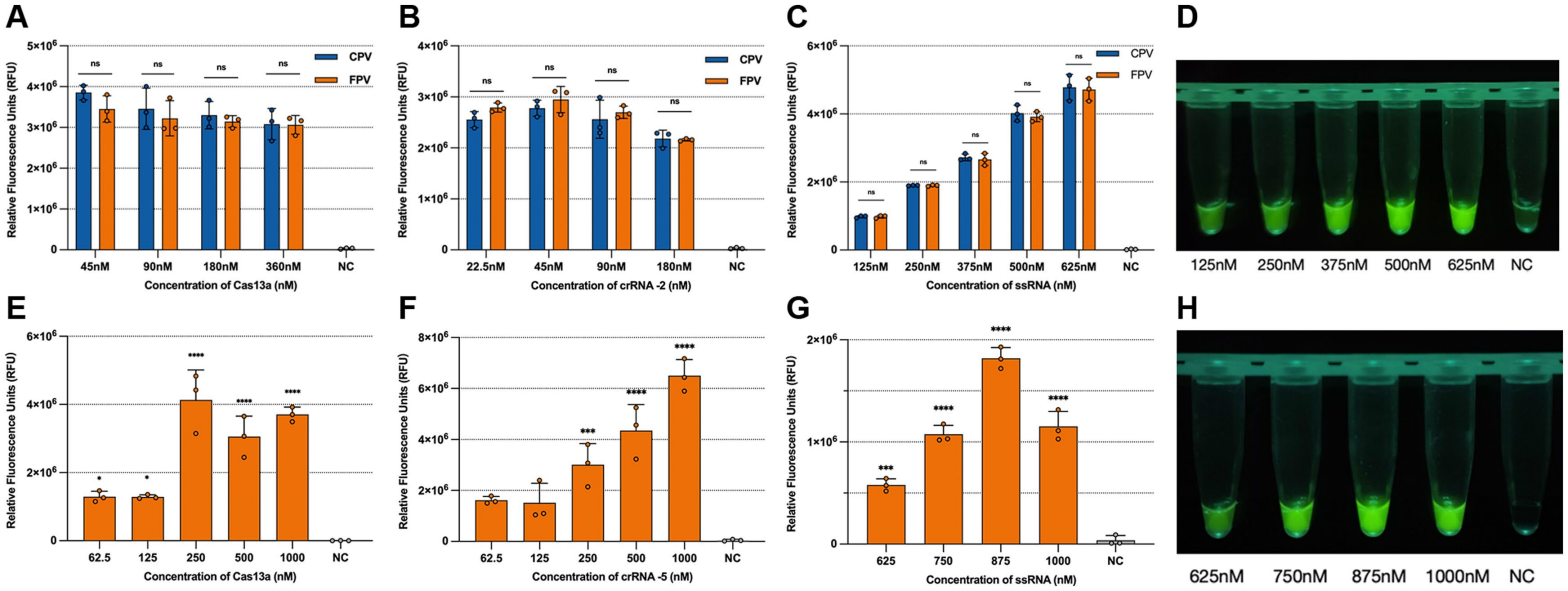
Optimization of the CRISPR/Cas13a system. Optimization of the universal detection CRISPR system: **(A)** Optimization of Cas13a concentration: 45 nM, 90 nM, 180 nM, and 360 nM; **(B)** Optimization of crRNA-2 concentration: 22.5, 45, 90, and 180 nM; **(C)** Optimization of ssRNA reporter concentration: 125, 250, 375, 500, and 625 nM; **(D)** Optimization of the visual result of the reporter under blue light. Optimization of the differentiation detection CRISPR system: **(E)** Optimization of Cas13a concentration: 62.5, 125, 250, 500, and 1,000 nM; **(F)** Optimization of crRNA-5 concentration: 62.5, 125, 250, 500, and 1,000 nM; **(G)** Optimization of ssRNA reporter concentration: 625, 750, 875, and 1,000 nM; **(H)** Optimization of the visual result of the reporter under blue light. The data are the mean ± s.d. for 3 technical replicates. ***p < 0.001, ****p < 0.0001.

To optimize detection performance, various reaction conditions were systematically evaluated based on the 30 min endpoint fluorescence intensity. For CPV-2 and FPV detection, the highest fluorescence signal was observed at a crRNA concentration of 45 nM; however, fluorescence intensities across different crRNA concentrations did not differ significantly. Similarly, a Cas13a concentration of 360 nM yielded the highest signal, though no statistically significant variation was observed among the tested concentrations. In contrast, fluorescence intensity increased proportionally with higher ssRNA reporter concentrations. To balance reagent usage with fluorescence visibility, the optimal conditions selected for subsequent detection assays were 22.5 nM crRNA, 45 nM Cas13a, and 375 nM ssRNA (Figures 4A–D). For differentiation between CPV-2 and FPV, the highest fluorescence intensity was obtained with 1,000 nM crRNA and 250 nM Cas13a. While increasing the ssRNA concentration initially enhanced fluorescence, the signal declined when ssRNA reached 1,000 nM. Accordingly, the optimized reaction conditions for the differentiation assay were set at 1000 nM crRNA, 50 nM Cas13a, and 875 nM ssRNA for subsequent experiments (Figures 4E–H).

### 3.4 Optimization of the one-tube RPA-CRISPR/Cas13a detection assay

**Figure 5:**
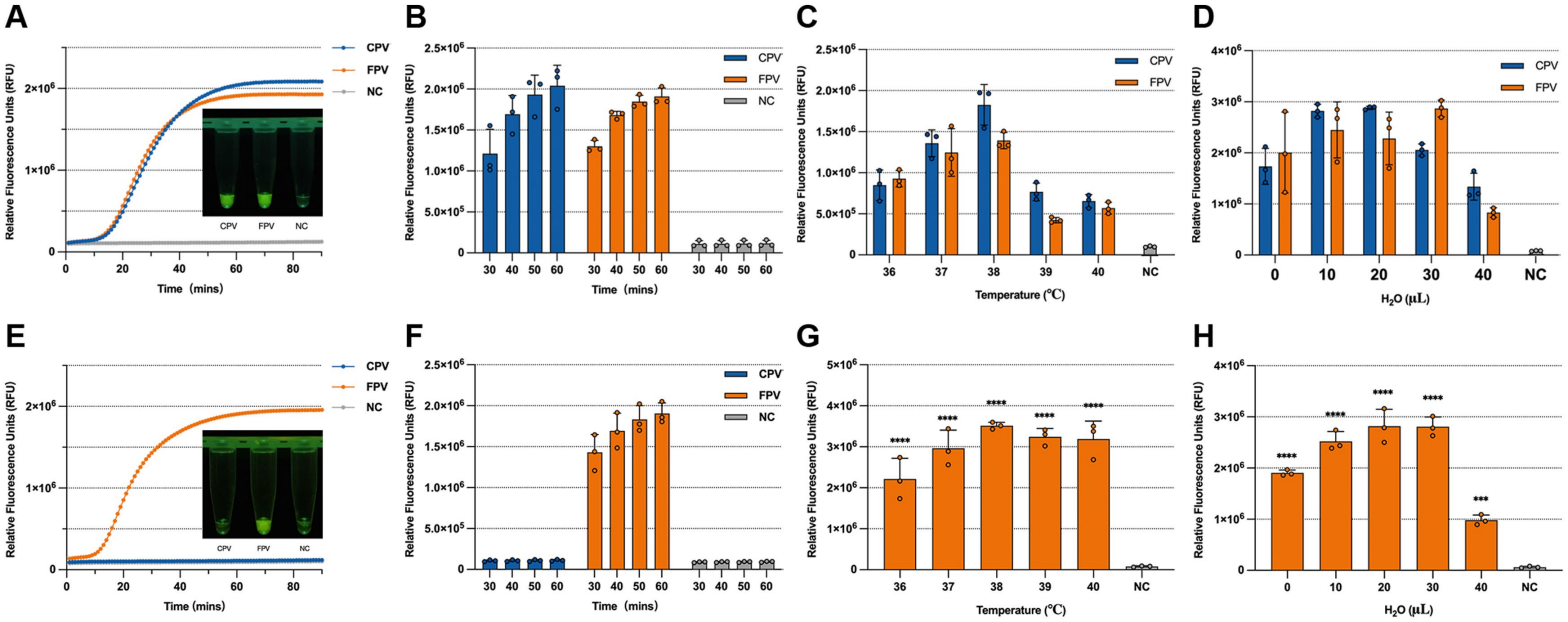
Optimization of the one-tube RPA-CRISPR/Cas13a detection method. **(A)** Fluorescence kinetics and visual results under blue light at 60 min for the universal detection system. **(B)** Endpoint fluorescence intensity of the universal detection system at 30, 40, 50, and 60 min. **(C)** Optimization of reaction temperature for the universal detection system. **(D)** Volume of dilution water used in the universal detection system. **(E)** Fluorescence kinetics and visual results under blue light at 60 min for the differentiation detection system. **(F)** Endpoint fluorescence intensity of the differentiation detection system at 30, 40, 50, and 60 min. **(G)** Optimization of reaction temperature for the differentiation detection system. **(H)** Volume of dilution water used in the differentiation detection system.

To improve the performance of the one-tube RPA-CRISPR/Cas13a assay, key reaction parameters including time, temperature, and reagent composition were systematically optimized. Evaluation of fluorescence intensity under different conditions revealed that a 60-min incubation at 38 °C produced the most consistent and visually distinguishable results in both detection and differentiation assays (Figures 5A–C,E). We defined visibly detectable fluorescence by the naked eye as positive; however, this criterion may involve a certain degree of subjectivity. To further quantify the detection limit, we analyzed the endpoint fluorescence signals from two detection systems and tested the blank control data, which showed no significant differences among groups (Supplementary Table S3). Based on the mean and standard deviation of these blank controls, the limit of detection (LLD = Mean+3SD) was estimated using a standard formula to be approximately 120,000, therefore, for both detection systems, fluorescence values exceeding 120,000 at 60 min are uniformly considered positive. Although shorter reaction times (e.g., 30 min) also generated detectable signals, the fluorescence intensity at 60 min was much stronger, suggesting higher sensitivity. Extending the reaction beyond 60 min did not result in a substantial increase in signal, indicating that longer incubation times were unnecessary. Therefore, a 60-min reaction was selected to ensure assay robustness. Additionally, when combining the RPA amplification and CRISPR detection steps into a single-tube format, a marked decrease in sensitivity was observed, potentially due to enzyme interference affecting T7 transcription (Arizti-Sanz et al., 2020). Drawing on previous findings that dilution can mitigate such effects (Li et al., 2023), we tested the addition of RNase-free ddH_2_O in varying volumes. A final addition of 20 μL RNase-free ddH_2_O was found to enhance fluorescence signal without compromising reaction efficiency and was thus chosen for subsequent experiments (Figures 5D,H).

### 3.5 Sensitivity of the RPA-CRISPR/Cas13a assay

**Figure 6:**
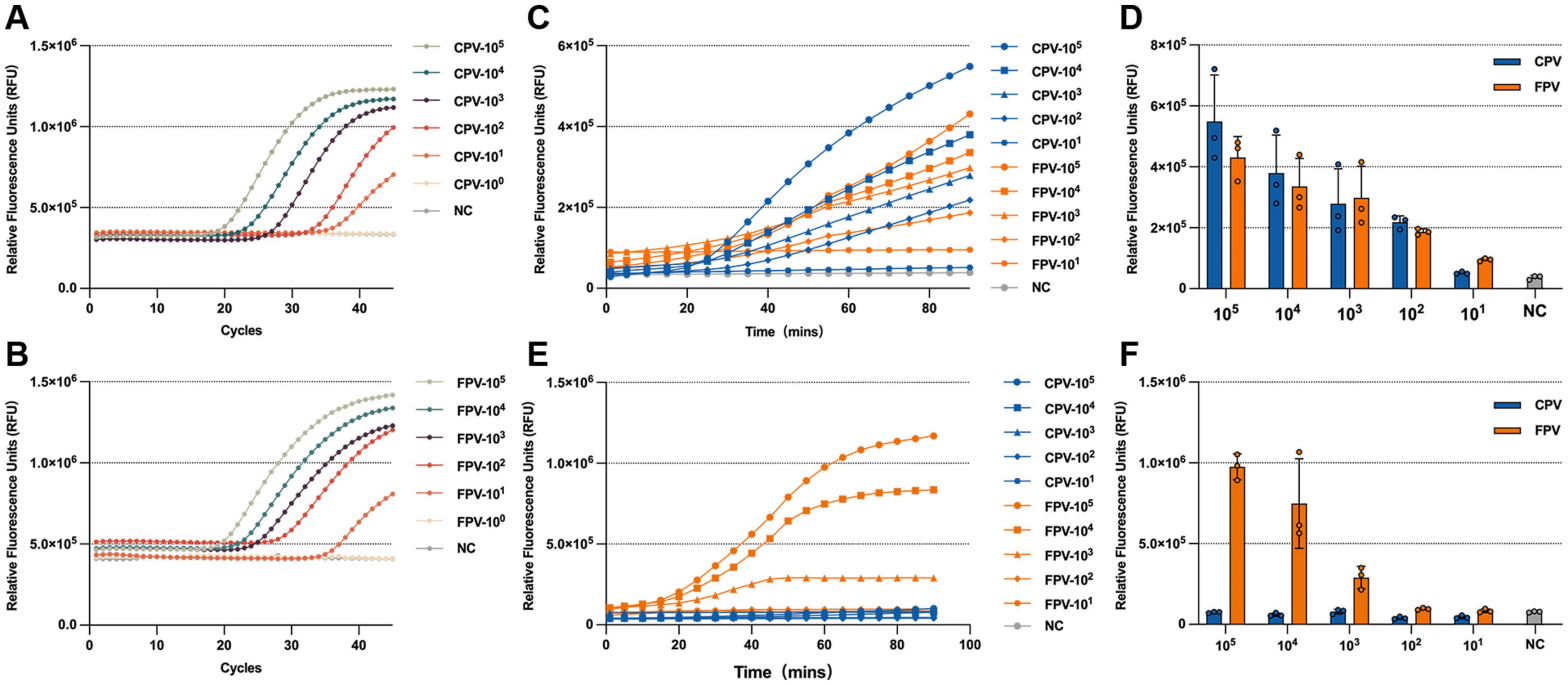
LOD of the RPA-CRISPR/Cas13a system. LOD of q-PCR for detecting CPV-2 **(A)** and FPV **(B)**, with samples considered positive when the Ct value is less than 35. Fluorescence kinetics **(C)** and endpoint fluorescence intensity at 60 min **(D)** for the LOD of the universal detection system. Fluorescence kinetics **(E)** and endpoint fluorescence intensity at 60 min **(F)** for the LOD of the differentiation detection system.

The copy number of the standard positive plasmid was calculated and serially diluted. Plasmid DNA ranging from (10^5^–10^1^ copies/μL) was used as a template to evaluate the LOD of the RPA-CRISPR/Cas13a detection assay. For comparison, q-PCR was also performed using the same template concentrations. The results showed that RPA-CRISPR/Cas13a system achieved a detection LOD of 10^2^ copies/μL for both CPV-2 and FPV VP2 genes (Figures 6C,D), which was slightly less sensitive than q-PCR, with a detection limit of 10^1^ copies/μL (Figures 6A,B). In the CPV-2 and FPV differentiation assay, significant FPV-specific fluorescence was observed at template concentrations as low as 10^3^ copies /μL, while CPV-2 fluorescence remained comparable to the negative control across all concentrations (Figures 6E,F).

### 3.6 Specificity of the RPA-CRISPR/Cas13a system

**Figure 7:**
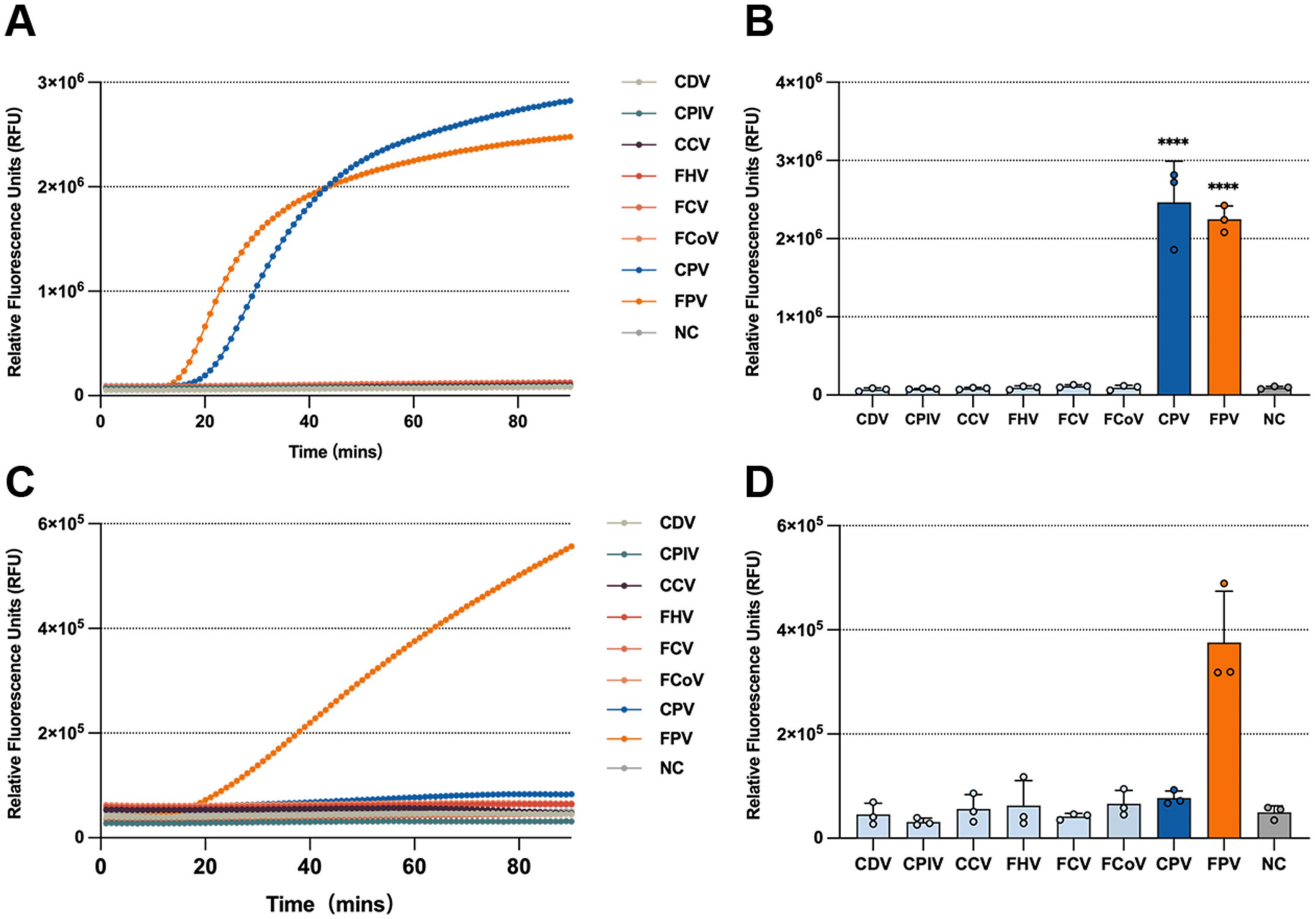
Specificity of the universal detection system and the differentiation detection system. Fluorescence kinetics **(A)** and endpoint fluorescence intensity at 60 min **(B)** of the universal detection system for different targets (CDV, CPIV, CCV, FHV, FCV, FCoV, CPV, and FPV). Fluorescence kinetics **(C)** and endpoint fluorescence intensity at 60 min **(D)** of the differentiation detection system for different targets (CDV, CPIV, CCV, FHV, FCV, FCoV, CPV, and FPV).

Specificity is a critical parameter for evaluating the performance of a diagnostic assay. To assess the specificity of the RPA-CRISPR/Cas13a-based detection system, nucleic acids from the following viruses were individually used as templates: canine distemper virus (CDV), canine coronavirus (CCV), canine parainfluenza virus (CPIV), feline coronavirus (FCoV), feline calicivirus (FCV), feline herpesvirus type 1 (FHV-1), CPV-2, and FPV. For the CPV-2 and FPV detection systems, only CPV-2 and FPV produced significantly elevated fluorescence signals, while the other six viruses generated signals comparable to the negative control (Figures 7A,B). Similarly, in the differentiation assay using crRNA-5 targeting the VP2 genes, only FPV produced a significantly higher fluorescence signal, whereas CPV-2 and all non-target viruses yielded minimal fluorescence (Figures 7C,D). These results demonstrate that the RPA-CRISPR/Cas13a-based assays possess high specificity for detecting and distinguishing CPV-2 and FPV.

### 3.7 Performance evaluation of the RPA-CRISPR/Cas13a system in clinical sample detection

To evaluate the clinical applicability of the RPA-CRISPR/Cas13a detection system, a total of 50 clinical samples were collected, including 25 canine-origin and 25 feline-origin samples. Nucleic acids extracted from these samples were tested using both q-PCR and the RPA-CRISPR/Cas13a assays. The detection results for the clinical samples are summarized in Table 1. According to the q-PCR results, 19 canine samples and 15 feline samples were positive, while the remaining samples were negative. The general RPA-CRISPR/Cas13a detection assay also identified 19 positive canine samples and 15 positive feline samples, fully consistent with the q-PCR results. Furthermore, the differential detection assay specifically identified all 15 FPV-positive samples, in agreement with the known origins of the samples. Overall, both the general and differential RPA-CRISPR/Cas13a assays demonstrated 100% concordance with q-PCR results, and were further validated by PCR and sequencing, indicating high accuracy and reliability for clinical diagnostics.

**Table 1:** Detection in clinical samples using the RPA-CRISPR/Cas13a-based universal and differentiation detection systems, as well as q-PCR and DNA sequencing.

RPA-CRISPR/Cas13a detection		qPCR or sequencing results		Concordance rate (%)	
A. Universal detection and qPCR (*n* = 50)					
		qPCR			
		+	−	Total	
Universal detection	Positive +	34	0	34	100
	Negative −	0	16	16	100
	Total	34	16	50	
B. Differential detection and DNA sequencing (on 34 positive samples)					
		DNA sequencing			
		CPV-2	FPV	Total	
Differentiation detection	CPV-2	0	15	15	100
	FPV	19	0	19	100
	Total	19	15	34	

## 4 Discussion

CPV-2 and FPV exhibit broad host tropism, with specific strains demonstrating cross-species transmission capabilities. These viruses are globally distributed, particularly the CPV-2c variant, which has been differentiated in wildlife populations across North America, Europe, Asia, and South America, emerging as the predominant circulating strain (Kimpston et al., 2022; Kolangath et al., 2023; Kurucay et al., 2023; Grecco et al., 2024). Notably, free-roaming domestic dogs and cats may serve as epidemiological bridges for parvovirus transmission, as their activity ranges often overlap with wildlife habitats, facilitating direct or indirect contact with wild carnivores and promoting cross-species viral spillover. Once introduced into immunologically naïve wildlife populations, these viruses can trigger outbreaks characterized by high juvenile mortality, posing serious conservation challenges to endangered species (Kelman et al., 2020; Kimpston et al., 2022). Currently, q-PCR serves as the gold standard for nucleic acid detection due to its high specificity and sensitivity. However, its reliance on specialized personnel, sophisticated equipment, and prolonged processing time limits field applicability. There is consequently an urgent need for rapid diagnostic alternatives with comparable accuracy. Therefore, there is a pressing need for diagnostic alternatives with comparable accuracy.

Independent studies have reported CRISPR-based detection methods for CPV-2 and FPV. Wang et al. developed an endpoint fluorescence assay based on RPA-Cas12a targeting the FPV NS1 gene, achieving a sensitivity of 1 copy/μL within 25 min (Wang et al., 2024). This method involves a two-step process: initial RPA amplification followed by the addition of the amplified product to the CRISPR/Cas12a system. When accounting for the time required for amplification and reagent transfer, the total detection time may be longer. Similarly, Khan et al. established a CRISPR-Cas13a nanoparticle detection system capable of identifying CPV-2 at a concentration of 100 amol/L within 30 min (Khan et al., 2019). The shorter detection time in this method may be attributed to differences in fluorescence signal measurement techniques and the criteria used to define a positive result. Although both methods offer advantages in terms of speed and cost-effectiveness, neither enables effective differentiation between CPV-2 and FPV. Current approaches for strain discrimination remain limited. Decaro et al. utilized minor groove binder (MGB) probes targeting a single nucleotide polymorphism (SNP) at position 3,753 in the viral genome for q-PCR-based differentiation (Decaro et al., 2008), whereas Sun et al. employed HRM analysis of SNP A4408C to generate distinct melting curves (Sun et al., 2019). Although accurate, these methods require advanced instrumentation and technical expertise, restricting their use in resource-limited settings.

In this study, we established a RPA-CRISPR/Cas13a platform capable of both detecting and differentiating CPV-2 and FPV. Unlike Wang et al., who targeted the NS1 gene for FPV detection (Wang et al., 2024), our study focused on the VP2 gene and developed a differentiation detection system based on mutation sites in the VP2 gene of CPV-2 and FPV to distinguish between the two pathogens. To enable virus differentiation, we systematically evaluated off-target effects of Cas13a by designing three mismatch-tolerant crRNAs, ultimately selecting optimal crRNA-RPA primer pairs. Through reaction optimization—including volume adjustment to mitigate RPA-mediated transcriptional inhibition—we developed a robust, one-tube detection protocol. The detection system achieved sensitivities of 10^2^ copies/μL for CPV-2 or FPV, while the differentiation system showed slightly reduced sensitivity (10^3^ copies/μL), likely due to limited tolerance of Cas13a for the single mismatch in crRNA-5. Although the sensitivity is slightly lower compared to some other studies, it fully meets the requirements for practical clinical detection. Both assays were completed within 60 min and exhibited exceptional specificity, showing no cross-reactivity with other common canine and feline pathogens, and were further validated using clinical samples with 100% concordance to qPCR and DNA sequencing results.

The practical applicability of our newly developed method is enhanced by its operational simplicity. The optimized one-tube method minimizes the risk of amplicon contamination by reducing manual handling steps. Furthermore, the results can be interpreted visually under blue light or with a basic fluorescence detector, making it highly suitable for point-of-care and field use.

It is worth noting that the number of samples and the diversity of species in this study were relatively limited, and no cases of co-infection with CPV-2 and FPV were identified among the collected specimens. Moreover, the detection method established in this study is currently unable to simultaneously detect both CPV-2 and FPV in co-infected samples, it can only identify the presence of FPV, while the detection of CPV-2 requires additional methods for confirmation. Future research should expand the range of target species and increase the sample size, while further optimizing the detection method to evaluate its effectiveness in distinguishing CPV-2 from FPV and its applicability in point-of-care testing. Additionally, improvements should aim to enable simultaneous detection of both CPV-2 and FPV in co-infected samples. From a cost-effectiveness perspective, the CRISPR/Cas13a detection method established holds significant potential for point-of-care tests (POCTs). For future translation and broader application, the core reaction can be adapted to later flow assay (Xiong et al., 2021) or integrated into automated microfluidic systems (Chen et al., 2023).

In summary, we have established a sensitive, specific, and user-friendly RPA-CRISPR/Cas13a platform for the rapid detection and differentiation of CPV-2 and FPV. By closely linking assay performance (sensitivity, specificity, speed, simplicity) to tangible field requirements, this work provides a promising diagnostic tool that can facilitate early outbreak detection, inform effective control strategies, and support conservation efforts, particularly in resource-constrained environments.

## Data Availability

The original contributions presented in the study are included in the article/Supplementary material, further inquiries can be directed to the corresponding authors.

## Footnotes

1
https://www.ncbi.nlm.nih.gov/
