# Supplementary material for: Rapid visual detection and differentiation of canine and feline parvovirus via a one-tube RPA-CRISPR/Cas13a assay
Source: Front Microbiol. 2025 Dec 19;16:1735549. doi: 10.3389/fmicb.2025.1735549 (PMC12757423; doi:10.3389/fmicb.2025.1735549)
Supplement: Supplementary file 1 [file Data_Sheet_1.docx]

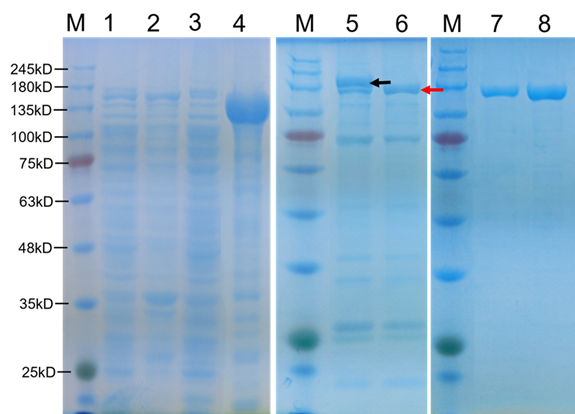


Supplementary Figure 1｜Expression and purification of of Cas13a protein. M: Protein molecular weight standard, 1: Cell lysate, 2: Supernatant of centrifuged cell lysate, 3: Pellet of centrifuged cell lysate, 4: 250 mM imidazole elution buffer, 5: Protein solution before Ulp1 digestion (black arrow indicates the SUMO-tagged LwCas13a protein), 6: Protein solution after Ulp1 digestion (red arrow indicates the LwCas13a protein with the SUMO tag removed), 7: Flow-through from Ni column loading of solution 6, 8: Purified LwCas13a (138kDa) in storage buffer.

**Supplementary Table 1**. The conserved regions of the CPV-2 and FPV VP2 genes that were inserted during the construction of recombinant plasmid standards.

| Name | Sequence (5′ → 3′) |
| --- | --- |
| CPV-VP2 | TGTGGGGATTTCTACGGGTACTTTCAATAATCAGACAGAATTTAAATTTTTGGAAAACGGATGGGTGGAAATCACAGCAAACTCAAGCAGACTTGTACATTTAAATATGCCAGAAAGTGAAAATTATAGAAGAGTGGTTGTAAATAATTTGGATAAAACTGCAGTTAACGGAAACATGGCTTTAGATGATACTCATGCACAAATTGTAACACCTTGGTCATTGGTTGATGCAAATGCTTGGGGAGTTTGGTTTAATCCAGGAGATTGGCAACTAATTGTTAATACTATGAGTGAGTTGCATTTAGTTAGTTTTGAACAAGAAATTTTTAATGTTGTTTTAAAGACTGTTTCAGAATCTGCTACTCAGCCACCAACTAAAGTTTATAATAATGATTTAACTGCATCATTGATGGTTGCATTAGATAGTAATAATACTATGCCATTTACTCCAGCAGCTATGAGATCTGAGACATTGGGTTTTTATCCATGGAAACCAACCATACCAACTCCATGGAGATATTATTTTCAATGGGATAGAACATTAATACCATCTCATACTGGAACTAGTGGCACACCAACAAATATATACCATGGTACAGATCCAGATGATGTTCAATTTTACACTATTGAAAATTCTGTGCCAGTACACTTACTAAGAACAGGTGATGAATTTGCTACAGGAACATTTTATTTTGATTGTAAACCATGTAGACTAACACATACATGGCAAACAAATAGAGCATTGGGCTTACCACCATTTCTAAATTCTTTGCCTCAAGCTGAAGGAGGTACTAACTTTGGTTATATAGGAGTTCAACAAGATAAAAGACGTGGTGTAACTCAAATGGGAAATACAAACATTATTACTGAAGCTACTATTATGAGACCAGCTGAGGTTGGTTATAGTGCACCATATTATTCTTTTGAGGCGTCTACACAAGGGCCATTTAAAACACCTATTGCAGCAGGACGGGGGGGAGCGCAAACAGATGAAAATCAAGCAGCAGATGGTGATCCAAGATATGCATTTGGTAGACAACATGGTCAAAAAACTACCACAACAGGAGAAACACCTGAGAGATTTACATATATAGCACATCAAGATACAGGAAGATATCCAGAAGGAGATTGGATTCAAAATATTAACTTTAACCTTCCTGTAACAAATGATAATGTATTGCTACCAACAGATCCAATTGGAGGTAAAACAGGAATTAACTATACTAATATATTTAATACTTATGGTCCTTTAACTGCATTAAATAATGTACCACCAGTTTATCCAAATGGTCAAATTTGGGATAAAGAATTTGATACTGACTTAAAACCAAGACTTCATGTAAATGCACCATTTGTT |
| FPV-VP2 | TGTGGGGATTTCTACGGGTACTTTCAATAATCAGACGGAATTTAAATTTTTGGAAAACGGGTGGGTGGAAATCACAGCAAACTCAAGCAGACTTGTACATTTAAATATGCCAGAAAGTGAAAATTATAAAAGAGTAGTTGTAAATAATATGGATAAAACTTCAGTTAAAGGAAACATGGCTTTAGATGATACTCATGTACAAATTGTAACACCTTGGTCATTGGTTGATGCAAATGCTTGGGGAGTTTGGTTTAATCCAGGAGATTGGCAACTAATTGTTAATACTATGAGTGAGTTGCATTTAGTTAGTTTTGAACAAGAAATTTTTAATGTTGTTTTAAAGACTGTTTCAGAATCTGCTACTCAGCCACCAACTAAAGTTTATAATAATGATTTAACTGCATCATTGATGGTTGCATTAGATAGTAATAATACTATGCCATTTACTCCAGCAGCTATGAGATCTGAGACATTGGGTTTTTATCCATGGAAACCAACCATACCAACTCCATGGAGATATTATTTTCAATGGGATAGAACATTAATACCATCCCATACTGGAACTAGTGGCACACCAACAAATGTATATCATGGTACAGATCCAGATGATGTTCAATTTTATACTATTGAAAATTCTGTACCAGTGCACTTACTAAGAACAGGTGATGAATTTGCTACAGGAACATTTTTTTTTGATTGTAAACCATGTAGACTAACACATACATGGCAAACAAATAGAGCATTGGGCTTACCACCATTTTTAAATTCTTTGCCTCAATCTGAAGGAGCTACTAACTTTGGTGATATAGGAGTTCAACAAGATAAAAGACGTGGTGTAACTCAAATGGGAAATACAGACTATATTACTGAAGCTACTATTATGAGACCAGCTGAGGTTGGTTATAGTGCACCATATTATTCTTTTGAAGCATCTACACAAGGGCCATTTAAAACACCTATTGCAGCAGGACGGGGGGGAGCGCAAACAGATGAAAATCAAGCAGCAGATGGTGATCCAAGATATGCATTTGGTAGACAACATGGTCAAAAAACTACTACAACAGGAGAAACACCTGAGAGATTTACATATATAGCACATCAAGATACAGGAAGATATCCAGAAGGAGATTGGATTCAAAATATTAACTTTAACCTTCCTGTAACAAATGATAATGTATTGCTACCAACAGATCCAATTGGAGGTAAAACAGGAATTAACTATACTAATATATTTAATACTTATGGTCCTTTAACTGCATTAAATAATGTACCACCAGTTTATCCAAATGGTCAAATTTGGGATAAAGAATTTGATACTGACTTAAAACCAAGACTTCATGTAAATGCACCATTTGTT |

The yellow highlighted regions indicate the target sites of the universal detection system, the green highlighted regions indicate the target sites of the differentiation detection system, and the red highlighted regions mark the mismatch positions between the crRNA of the differentiation detection system and the target sequence. The mismatch information between the two sequences is as follows: 37 (A→G), 61 (A→G), 129 (G→A), 136 (G→A), 149 (T→A), 161 (G→T), 169 (C→A), 198 (C→T), 553 (T→C), 584 (A→G), 589 (C→T), 622 (C→T), 640 (G→A), 646 (A→G), 690 (A→T), 761 (C→T), 779 (G→T), 789 (G→C), 803 (T→G), 857 (A→G), 860 (A→T), 861 (T→A), 928 (G→A), 931 (G→A), 1057 (C→T).

**Supplementary Table 2**. RPA-CRISPR/Cas13a and q-PCR assays primers and oligonucleotides.

| Name | Sequence (5′ → 3′) |  |
| --- | --- | --- |
| crRNA-F1 | GAAATTAATACGACTCACTATAGGGGATTTAGACTACCCCAAAAACGAAGGGGACTAAAACTTTATCCAAATGGTCAAATTTGGGATAA |  |
| crRNA-R1 | TTATCCCAAATTTGACCATTTGGATAAAGTTTTAGTCCCCTTCGTTTTTGGGGTAGTCTAAATCCCCTATAGTGAGTCGTATTAATTTC | |
| crRNA-F2 | GAAATTAATACGACTCACTATAGGGGATTTAGACTACCCCAAAAACGAAGGGGACTAAAACTGGTTATAGTGCACCATATTATTCTTTT | |
| crRNA-R2 | AAAAGAATAATATGGTGCACTATAACCAGTTTTAGTCCCCTTCGTTTTTGGGGTAGTCTAAATCCCCTATAGTGAGTCGTATTAATTTC | |
| crRNA-F3 | GAAATTAATACGACTCACTATAGGGGATTTAGACTACCCCAAAAACGAAGGGGACTAAAACAAATGCTTGGGGAGTTTGGTTTAATCCA | |
| crRNA-R3 | TGGATTAAACCAAACTCCCCAAGCATTTGTTTTAGTCCCCTTCGTTTTTGGGGTAGTCTAAATCCCCTATAGTGAGTCGTATTAATTTC | |
| crRNA-F4 | GAAATTAATACGACTCACTATAGGGGATTTAGACTACCCCAAAAACGAAGGGGACTAAAACGTCTTTTATAATTTTCACTTTCTGGCAT | |
| crRNA-R4 | ATGCCAGAAAGTGAAAATTATAAAAGACGTTTTAGTCCCCTTCGTTTTTGGGGTAGTCTAAATCCCCTATAGTGAGTCGTATTAATTTC | |
| crRNA-F5 | GAAATTAATACGACTCACTATAGGGGATTTAGACTACCCCAAAAACGAAGGGGACTAAAACCTCTTTAATAATTTTCACTTTCTGGCAT | |
| crRNA-R5 | ATGCCAGAAAGTGAAAATTATTAAAGAGGTTTTAGTCCCCTTCGTTTTTGGGGTAGTCTAAATCCCCTATAGTGAGTCGTATTAATTTC | |
| crRNA-F6 | GAAATTAATACGACTCACTATAGGGGATTTAGACTACCCCAAAAACGAAGGGGACTAAAACAAATGTATATCATGGTACAGATCCAGAT | |
| crRNA-R6 | ATCTGGATCTGTACCATGATATACATTTGTTTTAGTCCCCTTCGTTTTTGGGGTAGTCTAAATCCCCTATAGTGAGTCGTATTAATTTC | |
| RPA-F1 | CTGCATCATTGATGGTTGCATTAGATAG | |
| RPA-F2 | CTATGCCATTTACTCCAGCAGCTATGAG | |
| RPA-F3 | CATGGAAACCAACCATACCAACTCCATG | |
| RPA-F4 | TAATACGACTCACTATAGGGCACAGCAAACTCAAGCAGACTTGTACATTTAA | |
| RPA-F5 | TAATACGACTCACTATAGGGATAATCAGACGGAATTTAAATTTTTGGAAAAC | |
| RPA-F6 | TAATACGACTCACTATAGGGCTACGGGTACTTTCAATAATCAGACGGAATTT | |
| RPA-R1 | TAATACGACTCACTATAGGGCTACCAAATGCATATCTTGGATCACCAT | |
| RPA-R2 | TAATACGACTCACTATAGGGCATCTGCTGCTTGATTTTCATCTGTTTG | |
| RPA-R3 | TAATACGACTCACTATAGGGCCTGCTGCAATAGGTGTTTTAAATGGCC | |
| RPA-R4 | CCAATCTCCTGGATTAAACCAAACTCCCCAAGC | |
| RPA-R5 | CATAGTATTAACAATTAGTTGCCAATCTCCTG | |
| RPA-R6 | CTAAATGCAACTCACTCATAGTATTAACAATT | |
| ssRNA probe | FAM-rUrUrUrUrUrU-BHQ1 | |
| q-PCR-F | ACAGCAAACTCAAGCAGAC | |
| q-PCR-R | AACCAATGACCAAGGTGTTA | |
| q-PCR-probe | FAM-CTGCAGTTAACGGAAACATGGCTT-BHQ1 | |

**Supplementary Table 3.** Endpoint fluorescence values at 60 minutes for the blank controls of the universal detection system and the differential detection system.

| Group | Universal detection system | Differentiation detection system | | Mean |
| --- | --- | --- | --- | --- |
|  | 89185.64844 | 99303.5859375 |  | |
|  | 91784.078125 | 71848.90625 | |  |
|  | 70715.5390625 | 81745.90625 | |  |
| Mean | 83895.0885 | 84299.4661 | | 84097.2773 |
| SD | 11487.5303 | 13904.3288 | | 12695.9296 |

**Supplementary Table 4.** Endpoint fluorescence values of different concentrations of cas13a, crRNA, ssRNA, reaction time, temperature, and ddH2O content of the universal detection and the differential detection assays.

| **Optimization of Cas13a concentration (Figure 4A)** | | | | | | | | | | | | | | | | |
| --- | --- | --- | --- | --- | --- | --- | --- | --- | --- | --- | --- | --- | --- | --- | --- | --- |
| **Concentration (nM)** | **CPV** | | | | | | | | **FPV** | | | | | | | |
| 45 | 4024055.25 | | 3666120.5 | | | 3877839 | | | 3164524.75 | | 3801005 | | | | 3392371 | |
| 90 | 4001562.5 | | 2999236 | | | 3370219 | | | 2970881.5 | | 3721143.5 | | | | 2980609 | |
| 180 | 3220794.25 | | 3012938 | | | 3666365.5 | | | 3255950.75 | | 3195267.75 | | | | 2981541.75 | |
| 360 | 3148087.75 | | 2670440.75 | | | 3427112.5 | | | 3224765.25 | | 3163780.25 | | | | 2802386.75 | |
| NC | 13204.89 | | 40378.97 | | | 42868.29 | | |  | | | |  | | | |
| **Optimization of crRNA-2 concentration (Figure 4B)** | | | | | | | | | | | | | | | | |
| **Concentration (nM)** | **CPV** | | | | | | | | **FPV** | | | | | | | |
| 22.5 | 2696146.75 | | 2388575.75 | | | 2580648.75 | | | 2772152.25 | | 2888117.75 | | | | 2711927.5 | |
| 45 | 2919101.75 | | 2607898.25 | | | 2806741.5 | | | 3109712 | | 3084788 | | | | 2652976.25 | |
| 90 | 2293556.75 | | 2402468 | | | 2988365.75 | | | 2828517.75 | | 2668785 | | | | 2595097.5 | |
| 180 | 1992480.88 | | 2268611 | | | 2287640 | | | 2159850.5 | | 2182260.5 | | | | 2135840.5 | |
| NC | 22241.03 | | 48927.61 | | | 25471.51 | | |  | | | | | | | |
| **Optimization of ssRNA reporter concentration (Figure 4C)** | | | | | | | | | | | | | | | | |
| **Concentration (nM)** | **CPV** | | | | | | | | **FPV** | | | | | | | |
| 125 | 942959.5 | | 1000386.88 | | | 999161.5 | | | 932410.88 | | 1005940.44 | | | | 1001067 | |
| 250 | 1888115.13 | | 1902161.5 | | | 1901204.13 | | | 1874381.25 | | 1901258.63 | | | | 1912561.13 | |
| 375 | 2655699.5 | | 2676240.75 | | | 2837163 | | | 2486999.5 | | 2653881 | | | | 2844462 | |
| 500 | 3795497.75 | | 3998398.75 | | | 4269949 | | | 3784959.75 | | 3895315.5 | | | | 4070029.5 | |
| 625 | 4383609 | | 4829667.5 | | | 5136358 | | | 4377449.5 | | 4733167 | | | | 5040242 | |
| NC | 8809.57 | | 17593.10 | | | 26095.43 | | |  | | | | | | | |
| **Optimization of Cas13a concentration (Figure 4E)** | | | | | | | | | | | | | | | | |
| **Concentration (nM)** | | | | | **RFU** | | | | | | | | | | | |
| 62.5 | | | | | 1466879 | | | 1150479.38 | | | | 1263145.63 | | | | |
| 125 | | | | | 1261997 | | | 1242631 | | | | 1356721.25 | | | | |
| 250 | | | | | 3148621.75 | | | 4422715.5 | | | | 4830666 | | | | |
| 500 | | | | | 2445650.75 | | | 3066583.75 | | | | 3654226.5 | | | | |
| 1000 | | | | | 3493203 | | | 3921695.75 | | | | 3702393.75 | | | | |
| NC | | | | | 2059.34 | | | 1998.13 | | | | 6217.20 | | | | |
| **Optimization of crRNA-5 concentration (Figure 4F)** | | | | | | | | | | | | | | | | |
| **Concentration (nM)** | | | | | **RFU** | | | | | | | | | | | |
| 62.5 | | | | | 1786085 | | | 1562453.13 | | | | 1501490.63 | | | | |
| 125 | | | | | 2399183 | | | 1045284.19 | | | | 1098361 | | | | |
| 250 | | | | | 3801818.5 | | | 2145968.25 | | | | 3075374.25 | | | | |
| 500 | | | | | 5247131.5 | | | 3232456.5 | | | | 4560454.5 | | | | |
| 1000 | | | | | 7168413 | | | 5898585.5 | | | | 6434699.5 | | | | |
| NC | | | | | 28511.66 | | | 6912.38 | | | | 78391.39 | | | | |
| **Optimization of ssRNA reporter concentration (Figure 4G)** | | | | | | | | | | | | | | | | |
| **Concentration (nM)** | | | | | **RFU** | | | | | | | | | | | |
| 625 | | | | | 519955.5 | | | 642076.63 | | | | 574299.88 | | | | |
| 750 | | | | | 1030633.25 | | | 1019037.88 | | | | 1176385 | | | | |
| 875 | | | | | 1718401.88 | | | 1928357.88 | | | | 1812225.5 | | | | |
| 1000 | | | | | 1028671.25 | | | 1110999.13 | | | | 1315798.5 | | | | |
| NC | | | | | 9860.79 | | | 10792.81 | | | | 90888.97 | | | | |
| **Optimization of reaction temperature for the universal detection assay (Figure 5C)** | | | | | | | | | | | | | | | | |
| **Temperature (°C)** | **CPV** | | | | | | | | **FPV** | | | | | | | |
| 36 | 862931.06 | | 1026839.06 | | | 654767.25 | | | 899571.13 | | 1038668.56 | | | | 845464.56 | |
| 37 | 1465039.13 | | 1440054.75 | | | 1171635.38 | | | 1170977.25 | | 1568002.63 | | | | 1004024.25 | |
| 38 | 1542149.13 | | 1964012.75 | | | 1974040.5 | | | 1335250.44 | | 1505717.75 | | | | 1334277.38 | |
| 39 | 675339.5 | | 740900.56 | | | 885239.5 | | | 459341.75 | | 414665.63 | | | | 396644.19 | |
| 40 | 669252.69 | | 726294.31 | | | 567097.56 | | | 557487.81 | | 645464.56 | | | | 503830.25 | |
| NC | 100637.95 | | 118418.77 | | | 104274.91 | | |  | | | | | | | |
| **Volume of dilution water used in the universal detection assay (Figure 5D)** | | | | | | | | | | | | | | | | |
| **ddH_2_O (μL)** | **CPV** | | | | | | | | **FPV** | | | | | | | |
| 0 | 1422509.13 | | 2117205.75 | | | 1668838.75 | | | 2810581.75 | | 1980057.88 | | | | 1229103.25 | |
| 10 | 2954452.25 | | 2698775.25 | | | 2815364.5 | | | 2848523.25 | | 2676529.25 | | | | 1823258 | |
| 20 | 2897003.25 | | 2899102 | | | 2844822.5 | | | 2662541.75 | | 2488035.5 | | | | 1698544.5 | |
| 30 | 2058262 | | 2177187 | | | 1943681.38 | | | 2882489.75 | | 3024519 | | | | 2696662.38 | |
| 40 | 1643544.25 | | 1171983.5 | | | 1200278.88 | | | 831370.31 | | 738738.19 | | | | 923258 | |
| NC | 80270.94 | | 78908.23 | | | 67020.71 | | |  | | | | | | | |
| **Optimization of reaction temperature for the differentiation detection assay (Figure 5G)** | | | | | | | | | | | | | | | | |
| **Temperature (°C)** | | | | | **RFU** | | | | | | | | | | | |
| 36 | | | | | 1735839.63 | | | 2163597 | | | | 2741401.25 | | | | |
| 37 | | | | | 2559507.25 | | | 2891447.75 | | | | 3437964.25 | | | | |
| 38 | | | | | 3432661.25 | | | 3598215 | | | | 3515524.25 | | | | |
| 39 | | | | | 3017514.5 | | | 3302875 | | | | 3410656.5 | | | | |
| 40 | | | | | 2684872.25 | | | 3377728 | | | | 3500368.25 | | | | |
| NC | | | | | 82924.58 | | | 61134.20 | | | | 91646.87 | | | | |
| **Volume of dilution water used in the differentiation detection assay (Figure 5H)** | | | | | | | | | | | | | | | | |
| **ddH_2_O (μL)** | | | | | **RFU** | | | | | | | | | | | |
| 0 | | | | | 1861898 | | | 1885142.5 | | | | 1969480.5 | | | | |
| 10 | | | | | 2741549.25 | | | 2437692.5 | | | | 2389708 | | | | |
| 20 | | | | | 2500520.5 | | | 2788880 | | | | 3162834.5 | | | | |
| 30 | | | | | 2632757.5 | | | 2780289.75 | | | | 3011679.25 | | | | |
| 40 | | | | | 962031.38 | | | 895842.88 | | | | 1091847.38 | | | | |
| NC | | | | | 80411.78 | | | 59949.49 | | | | 48531.22 | | | | |
| **Endpoint fluorescence values of the universal detection assay at different time (Figure 5B)** | | | | | | | | | | | | | | | | |
| **Time**  **(min)** | **CPV** | | | | | **FPV** | | | | | **NTC** | | | | | |
| 30 | 1014218.13 | 1066187.88 | | 1553977.88 | | 1251113.38 | 1267246 | | | 1384490.5 | 155271.56 | | | 87534.63 | | 88231.57 |
| 40 | 1450731.88 | 1720066.5 | | 1908000.88 | | 1631872.25 | 1717902.13 | | | 1700185.75 | 156402.67 | | | 89678.27 | | 89756.30 |
| 50 | 1658684.5 | 2058938.88 | | 2079043.88 | | 1789626.25 | 1934360.63 | | | 1817116.25 | 158135.39 | | | 91816.63 | | 91168.08 |
| 60 | 1752701 | 2222852.5 | | 2143935.5 | | 1845867.13 | 2030024.5 | | | 1849836.75 | 159880.13 | | | 94297.15 | | 92928.52 |
| **Endpoint fluorescence values of the universal detection assay at different time (Figure 5F)** | | | | | | | | | | | | | | | | |
| **Time (min)** | **CPV** | | | | | **FPV** | | | | | **NTC** | | | | | |
| 30 | 94643.24 | 104633.20 | | 115292.84 | | 1438372.25 | 1644355 | | | 1207252 | 81979.63 | | | 92058.58 | | 93298.39 |
| 40 | 96072.29 | 106221.70 | | 117579.02 | | 1678047.88 | 1916058.63 | | | 1484494.25 | 83390.25 | | | 93767.81 | | 94936.25 |
| 50 | 97850.83 | 107698.43 | | 119722.21 | | 1775756.5 | 2021287.63 | | | 1699309.25 | 85108.01 | | | 95292.86 | | 96726.13 |
| 60 | 100231.74 | 109324.92 | | 122545.49 | | 1804852 | 2051384.25 | | | 1856911.5 | 87238.78 | | | 97437.89 | | 98707.21 |
